# Mavacamten as an Effective Treatment for HCM With Latent Exercise‐Induced Obstruction: A Case Report

**DOI:** 10.1155/cric/9385301

**Published:** 2026-04-17

**Authors:** Nikitha Murthy, Brian Diep, Jonathan Evans, Dmitry Abramov, Diane Tran

**Affiliations:** ^1^ Division of Cardiology, Loma Linda University Health, Loma Linda, California, USA, lluh.org

**Keywords:** hypertrophic cardiomyopathy, mavacamten, treadmill stress echocardiography, Valsalva

## Abstract

**Background:**

Hypertrophic cardiomyopathy (HCM) is a myocardial disorder characterized by hypertrophy of the left ventricle that can lead to significant symptoms, particularly with exercise. For patients with HCM who demonstrate obstructive physiology, the first‐in‐class targeted cardiac specific myosin inhibitor, mavacamten, has been found to reduce the degree of obstruction as well as symptoms.

**Case Summary:**

We present a patient with symptomatic HCM who had no left ventricular outflow tract obstruction (LVOTO) at rest but demonstrated obstruction on treadmill stress echocardiography. She had symptomatic improvement after treatment with mavacamten.

**Discussion:**

Patients with symptomatic HCM who do not demonstrate elevated resting gradients may benefit from evaluation of exercise‐induced gradients to diagnose obstructive HCM and guide initiation of targeted therapy with mavacamten.

**Take Home Message:**

Mavacamten may be safe and effective in patients who demonstrate LVOTO only upon provocation with exercise.

## 1. Introduction

Hypertrophic cardiomyopathy (HCM) is a myocardial disorder characterized by hypertrophy of the left ventricle, which can consequently lead to a range of symptoms. It is a complex disease that greatly affects cardiac physiology including hypercontractility, diastolic abnormalities, dynamic left ventricular outlet obstruction (LVOTO), mitral valvular abnormalities, and arrhythmias [1].

Diagnosis of HCM is mainly based on identification of increased left ventricular wall thickness not attributed to other cardiac or systemic conditions. The standard threshold for diagnosis is an end‐diastolic wall thickness of 15 mm or greater anywhere in the left ventricle (or 13 mm or greater in those with a family history of HCM) as measured by transthoracic echocardiography (TTE) or cardiac magnetic resonance (CMR). Continuous wave Doppler interrogation by TTE is suggested for evaluation of obstructive physiology. A peak instantaneous continuous‐wave Doppler gradient of ≥ 30 mmHg at rest or > 50 mmHg with provokable measures indicates severe LVOTO and should prompt consideration of therapy in symptomatic individuals [2]. In patients with symptomatic HCM who do not have resting or maneuver–provokable gradients diagnostic of obstruction, exercise treadmill stress echocardiography (TSE) is recommended for further detection of latent LVOTO [3]. Given the dynamic nature of LVOTO, the use of TSE has shown promising diagnostic value in classifying obstructive HCM as these patients are otherwise underdiagnosed and suboptimally treated.

LVOTO is influenced by LV preload, myocardial contractility, and LV afterload. As such, medical therapy for symptomatic obstructive HCM is directed toward these targets. Medications include nonvasodilating beta‐blockers, nondihydropyridine calcium channel blockers, and if symptoms persist, the addition of disopyramide in combination with one of the prior medications [2]. In patients unresponsive to medical management or with severely elevated gradients, invasive options like septal reduction therapy can be considered [2].

Mavacamten, a first‐in‐class targeted cardiac‐specific myosin inhibitor, has emerged as a novel treatment option for obstructive HCM. Mavacamten was approved after several prospective studies in patients with obstructive HCM, both at rest and post‐exercise, demonstrated improvement in LVOT gradient, New York Heart Association (NYHA) II and III functional class, and exercise functional capacity [4–7]. Based on these studies, mavacamten was approved by the FDA and is indicated in patients with symptomatic NYHA Classes II and III obstructive HCM [5].

We present a patient with symptomatic NYHA Class III HCM who did not have an elevated LVOT gradient at rest or with Valsalva on initial TTE. After TSE, her peak gradient was diagnostic of obstructive HCM and she was subsequently treated with mavacamten with prompt improvement in symptoms.

## 2. Case Presentation

**Figure 1:**
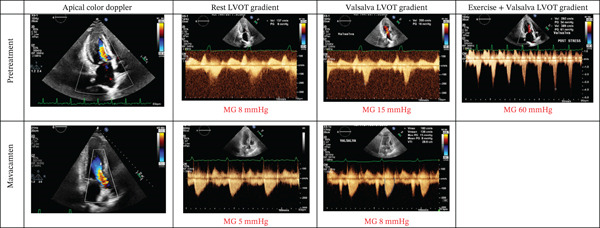
Transthoracic echocardiographic images demonstrating increased LVOT gradient with both exercise and Valsalva maneuver and incremental improvement after treatment with mavacamten.

The patient is a 45‐year‐old female with a known history of HCM with ICD implantation due to a history of syncope, nonsustained ventricular tachycardia (NSVT), and > 15% of scar burden on cardiac MRI who had several emergency department and clinic visits for chest pain as well as NYHA Class III symptoms, particularly dyspnea on exertion. She was on maximally tolerated metoprolol succinate and diltiazem, but still experienced exertional dyspnea. Her initial TTE was remarkable for no significant LVOT gradient at rest (4 mmHg) as well as the absence of systolic anterior motion (SAM). Two weeks later, she had her TSE, which demonstrated a post‐exercise LVOT peak gradient of 25 mmHg and a post‐exercise Valsalva LVOT peak gradient of 60 mmHg (Figure 1). Additionally, moderate SAM with moderate mitral regurgitation was provoked after exercise. She started on mavacamten 5 mg and was continued on the same doses of metoprolol succinate and diltiazem. One month after mavacamten initiation, her peak gradient with Valsalva remained low at < 10 mmHg and SAM was no longer seen (Figure 1). Her second‐month follow‐up TTE revealed similar findings. She no longer experienced chest pain and had significant improvement in her dyspnea on exertion, with NYHA classification reduced from III to I.

## 3. Discussion

This case report highlights a patient with symptomatic obstructive HCM who had no significant outflow gradient at rest but who demonstrated a prominent increase in LVOT gradient during TSE, particularly during TSE with Valsalva. Because of the provoked gradient, she was subsequently started on mavacamten with significant improvement in symptoms. This case report demonstrates the importance of screening symptomatic HCM patients for exercise‐induced gradients, as such patients may derive significant symptomatic benefit from mavacamten initiation.

Exercise‐induced LVOTO may be clinically relevant in patients without a baseline gradient for several reasons. Increase in heart rate and contractility with exercise leads to decreased diastolic filling time subsequently worsening SAM and exercise‐induced mitral regurgitation. This may lead to higher left atrial pressure, and therefore dyspnea or exertion or other exertional symptoms associated with increased left atrial or left ventricular end‐diastolic pressure. Exercise‐induced changes in hemodynamics may therefore be the main driver of exertional symptoms in a cohort of patients without resting obstruction, and these patients may benefit from mavacamten therapy [8].

Mavacamten reduces LVOTO through its effects on hypercontractility. Mavacamten acts as an allosteric inhibitor of cardiac myosin ATPase, thereby reducing actin–myosin cross‐bridge formation, reducing sarcomere power, and ultimately decreasing contractility and resolving SAM. Beyond contractility, mavacamten has beneficial effects on cardiac remodeling by reducing left ventricular mass and wall thickness [9]. The safety profile of mavacamten is generally favorable; however, given its mechanism of action, the primary safety concern lies in the drug′s ability to decrease left ventricular ejection fraction (LVEF). Thus, it is only recommended for use in those with a LVEF > 50%. Of note, in the landmark EXPLORER‐HCM trial, all LVEF reductions were reversible upon treatment interruption [5][10].

Our patient demonstrated improvement in symptoms and NYHA class by two classes, as consistent with published data on the symptomatic benefits of mavacamten [5–7]. These findings demonstrate the key concept that HCM physiology is dynamic. As such, the evaluation of exercise‐induced hemodynamics may increase the number of HCM patients who may currently be candidates for mavacamten therapy as compared to relying solely on resting gradient assessment. Additional studies will be needed on the patient selection for mavacamten using exercise or other hemodynamic parameters.

## 4. Conclusion

Mavacamten may be safe and effective in patients who only demonstrate LVOTO upon provocation with TSE. The population of HCM patients who demonstrate inducible LVOTO deserves further evaluation, including to best identify and risk‐stratify patients who may benefit from mavacamten or other myosin inhibitors under development.

## Funding

No funding was received for this manuscript.

## Ethics Statement

Informed consent was secured from the patient for all treatments and procedures.

## Consent

No written consent has been obtained from the patient as there are no patient identifiable data included in this case report.

## Conflicts of Interest

The authors declare no conflicts of interest.

## Data Availability

Data sharing not applicable to this article as no datasets were generated or analyzed during the current study.
